# Depletion of macrophages during early postnatal development leads to disrupted tooth root development and altered Gli1⁺ MSC trajectory

**DOI:** 10.1038/s41419-026-08753-7

**Published:** 2026-04-26

**Authors:** Yan Lei, Wen Li, Shiqing Wang, Lina Zhao, Yue Liu, Tingting Wang, Junjie Jiang, Xiao Su, Yufang Shi, Changshun Shao, Peishan Li

**Affiliations:** 1 The Fourth Affiliated Hospital of Soochow University, State Key Laboratory of Radiation Medicine and Protection, Institutes for Translational Medicine, Suzhou Medical College of Soochow University, Suzhou, China; 2 Biomedical Basic Research Center (BBRC) of Jiangsu Province, Suzhou, China

**Keywords:** Differentiation, Mesenchymal stem cells

## Abstract

Mammalian tooth development progresses through two principal stages-crown formation and root development-orchestrated by intricate interactions between the oral epithelium and neural crest-derived mesenchyme. After crown formation, Hertwig’s epithelial root sheath (HERS) directs root development. In this phase, Gli1⁺ mesenchymal stem cells (MSCs) give rise to dental pulp, dentin, cementum, and the periodontal ligament (PDL). The root anchors the tooth to the alveolar bone via PDL fibers, forming a dynamic occlusal buffer that mediates mechanosensation and nutrient supply. Although previous work has shown that macrophages are abundant in the dental pulp and follicle, the functional importance of macrophages in tooth development has not been well characterized. Here, we investigated the spatiotemporal dynamics of macrophage populations (identified by CD68, F4/80, CD206, and other markers) in molars and surrounding tissues during postnatal root development in mice. Importantly, Macrophage depletion via clodronate liposomes resulted in shortened root, impaired PDL elongation and retarded alveolar bone shooting surrounding the root. Gli1⁺ MSCs exhibited increased proliferation but impaired osteo/odontogenic differentiation upon macrophage depletion. Single-cell RNA sequencing and in vitro co-culture experiments support a model in which macrophage-derived TGF-β acts on mesenchymal TGF-β receptors to direct MSC fate and thereby regulate root morphogenesis. Collectively, these findings establish macrophages as critical niche components that orchestrate tooth root development through immune–mesenchymal crosstalk.

## Introduction

Mammalian teeth originate from the oral epithelium and neural crest-derived mesenchyme, and their development progresses through two key stages: crown formation and root development [1]. Tooth development is a highly orchestrated process that initiates with the thickening of the oral epithelium. As embryogenesis progresses, the reciprocal interactions between the oral epithelium and the underlying mesenchyme intensify. Through precise and concerted regulation, the tooth germ sequentially progresses through the bud, cap, and bell stages. In the late bell stage, ameloblasts and odontoblasts differentiate, depositing enamel and dentin matrices, respectively, ultimately resulting in crown formation [2]. Following completion of crown development, root formation is initiated alongside periodontal tissue development and continues until tooth eruption [3].

The tooth root, serving as the *hidden pillar* of the tooth, anchors the tooth within the alveolar bone through fibrous connections of the PDL. The tooth and jawbone form a dynamic occlusal buffering system that enables the tooth to withstand masticatory forces [4]. Additionally, the root contains abundant neural, vascular, and lymphatic tissues that connect to the vasculature within the jawbone through the apical foramen, facilitating mechanosensation and nutrient supply [5]. Clinically, root developmental defects-such as short-root anomaly, root resorption, or defective cementogenesis-often lead to premature tooth loss, pulpal disease, and occlusal dysfunction, severely compromising oral health and quality of life [3]. Therefore, the root is an essential structural and functional component of the tooth, crucial for sustaining tooth stability and oral health.

Root development, like crown formation, depends on continuous epithelial–mesenchymal interactions, as well as the integration of root structures with the surrounding jawbone, vascular supply, and neural innervation [6, 7]. After crown completion, the proliferative edge of the cervical loop gives rise to a bi-layered epithelial structure known as Hertwig’s epithelial root sheath (HERS), which is composed of inner and outer enamel epithelium. HERS elongates apically and guides root formation by determining the size, shape, and number of roots [8, 9]. Under the guidance of HERS, Gli1⁺ mesenchymal stem cells (MSCs) residing in the apical region of the roots function as key progenitor cells during root morphogenesis. Via self-renewal and multilineage differentiation, Gli1⁺ MSCs contribute to the formation of dental pulp, dentin, cementum, PDL, and alveolar bone. Their spatiotemporally regulated differentiation and mineralization are critical for determining the structural integrity of developing roots [4, 10, 11].

Recent studies have revealed that tissue development is intricately regulated by the local microenvironment [12, 13]. As key elements of the innate immune system, macrophages exert multifaceted regulatory functions that extend well beyond conventional immunological responses [14–16]. As early as 1987, Jontell et al. identified Leu-M5^+^ macrophages (CD11c^+^) in healthy human dental pulp [17]. Later research revealed their preferential localization within the dentin and subodontoblastic regions during physiological root resorption of deciduous teeth, where their cellular processes infiltrate dentinal tubules [18, 19]. Animal studies further confirmed the presence of macrophages in the dental pulp of developing mouse molars and aged rat molars [20, 21]. Immunohistochemical analysis identified F4/80^+^ macrophages enriched in the dental pulp and follicle throughout molar morphogenesis in mice [22]. Although both human and mouse teeth contain abundant macrophages [20, 23], their specific roles in tooth morphogenesis remain unclear. Notably, *Csf1r*-deficient mice exhibit osteopetrosis and failed tooth eruption due to the loss of macrophages [24]. Moreover, in dental pulp injury models, macrophages expressing lymphatic vessel endothelial hyaluronan receptor-1 (LYVE-1) infiltrate the neoformed odontoblastic layer, where they secrete matrix metalloproteinases (MMPs) and pro-angiogenic growth factors to facilitate vascularization [25]. Macrophage depletion significantly reduces dental pulp stem cell (DPSC) populations, increases neutrophil infiltration, exacerbates local inflammation, and impairs reparative dentinogenesis in these models [26]. Collectively, these findings indicate a critical involvement of macrophages in odontogenesis and dental regenerative processes.

The transforming growth factor-β (TGF-β) signaling pathway serves as a master regulator orchestrating organogenesis and tissue homeostasis and plays an indispensable role in root morphogenesis [1]. Studies have shown that TGF-β signaling, through Smad-dependent pathways mediated by TGF-β type I/II receptors, precisely regulates epithelial–mesenchymal interactions, cell fate determination, and mineralized tissue formation during root development [27, 28]. Genetic ablation of TGF-β receptors in dental mesenchymal cells disrupts this finely tuned regulatory network and results in root developmental defects, including shortened roots, deficient cementum formation, and disorganized periodontal structures [29, 30]. However, the origin of TGF-β ligands and their temporospatial regulation within the root microenvironment remains poorly understood.

In this study, selective depletion of macrophages during early postnatal development led to pronounced morphological and functional defects, including shortened roots, impaired PDL elongation and retarded alveolar bone formation, which was attributed to disrupted mesenchymal differentiation. Indeed, in the absence of macrophages, Gli1⁺ MSCs tended to expand proliferatively at the expense of osteo-/odontogenic differentiation. Single-cell transcriptomics revealed that macrophages may direct MSC lineage commitment via TGF-β signaling, which is supported by in vitro assays showing that macrophage-derived cues promote osteogenic differentiation.

## Results

### Dynamic distributions of macrophages during early root development of mouse molars

**Fig. 1:**
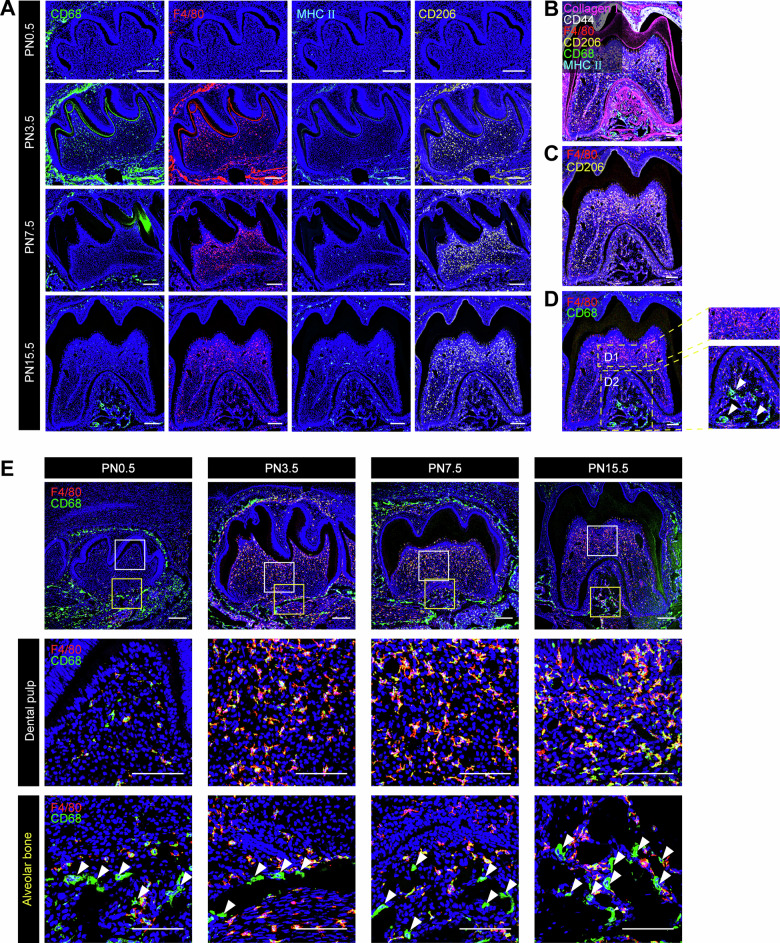
Spatial distribution of macrophages during molar root development. **A** Mass cytometry profiling of surface markers on macrophages from first mandibular molars from PN 0.5 to 15.5. **B** Multiplex immunostaining of the PN15.5 molar showing collagen I enrichment in dentin and bone matrices, and CD44 expression predominantly in the epithelium. **C** Co-staining of F4/80 (red) and CD206 (yellow) in first mandibular molars at PN15.5. **D** Co-staining of F4/80 (red) and CD68 (green); boxed areas are shown at higher magnification. White arrows indicate F4/80^-^CD68^+^ cells; D1 highlights the pulp region, and D2 highlights the furcation region. **E** Immunofluorescence co-staining of F4/80 (red) and CD68 (green); white arrows indicate F4/80^-^CD68^+^cells. The white box shows the pulp region, and the yellow box shows the alveolar bone. Scale bars: 100 μm.

Teeth comprise diverse cellular populations organized into specialized structures. Flow cytometry analysis of mandibular first molars and surrounding tissues identified that macrophages constitute the predominant immune cell population in the molar (Fig. S1A). To further explore immune cell dynamics during root development, we performed imaging mass cytometry on mandibular first molars at postnatal days 0.5, 3.5,7.5 and 15.5 (PN0.5-15.5). We found prominent colonization of CD68^+^F4/80^+^ macrophages in the coronal pulp region, an active odontogenic zone at PN0.5 (Fig. 1A), suggesting their participation in dentin formation [31]. At PN3.5, F4/80⁺CD68⁺ macrophages robustly accumulated in the dental follicle and pulp (Fig. 1A). With advancing root development, their number increased and distribution expanded throughout the pulp and periodontal ligament (Figs. 1A and S1B). In contrast, neutrophils (Ly6G⁺) localized predominantly in the alveolar bone marrow space (Fig. S1C), while CD3⁺ T cells were scarce and primarily confined to the marrow space (Fig. S1D).

The macrophage population exhibits heterogeneity across root development. F4/80⁺CD68⁺ macrophages predominantly displayed an M2-like phenotype (CD206⁺, Fig. 1B, C), suggesting potential involvement in root morphogenesis through trophic signaling [32]. The CD68^+^F4/80^-^ osteoclasts (TRAP⁺ multinucleated cells) were identified within the dental follicle and alveolar bone (Figs. 1D, E and S1E), actively contributing to alveolar bone remodeling to generate the biomechanical force required for tooth eruption. These phenotypically heterogeneous macrophages play distinct roles in maintaining tissue homeostasis during tooth development.

### Depletion of macrophages leads to retarded root growth

**Fig. 2:**
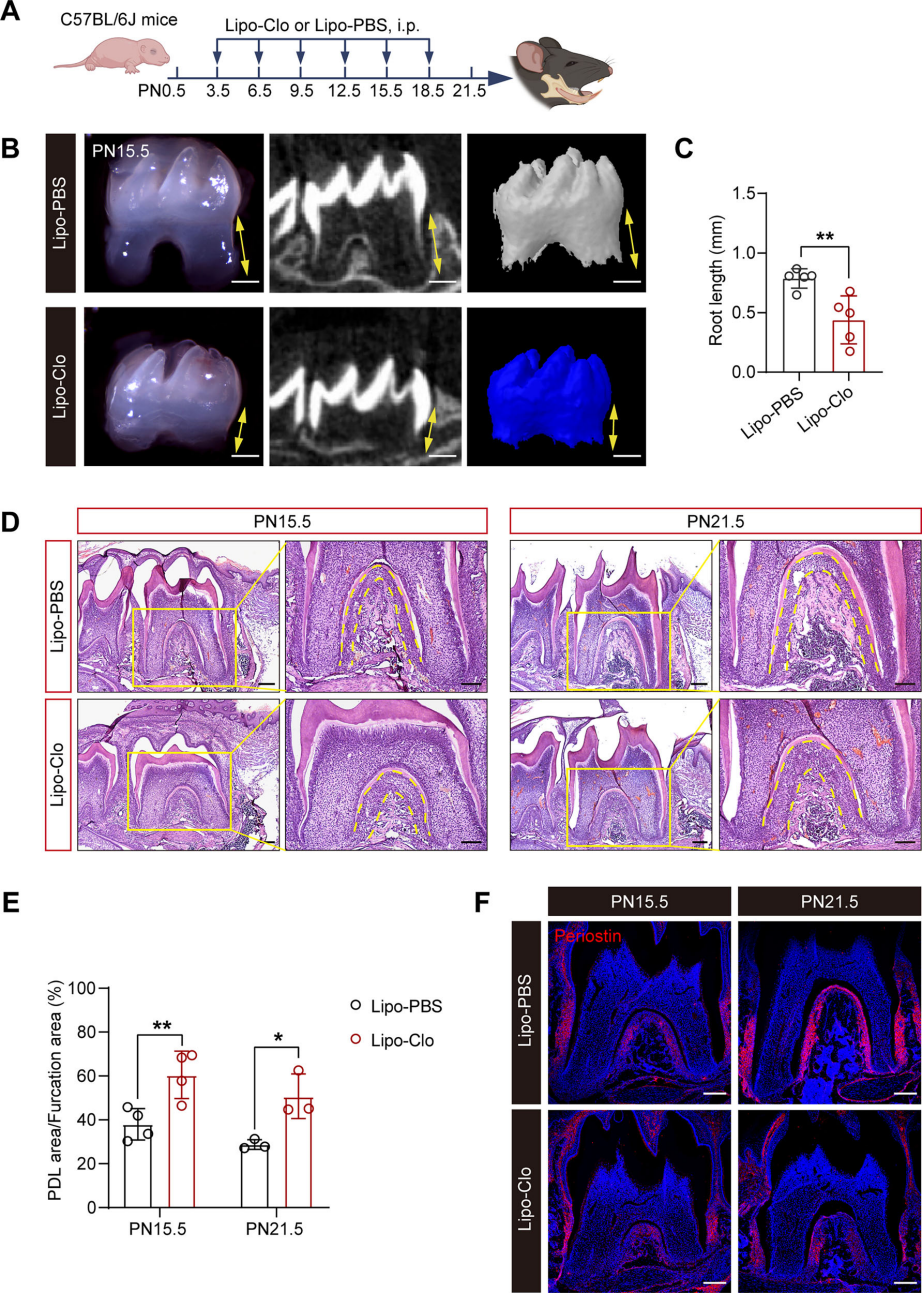
Macrophage depletion disrupts molar root development and periodontal integrity. **A** Diagram of the experimental procedure for macrophage depletion with clodronate liposomes. **B** Representative photographs, 2D, and 3D micro-CT images of first mandibular molars in Lipo-PBS- and Lipo-Clo-treated mice at PN15.5. Double arrowheads indicate root length. **C** Quantification of root length at PN15.5 (*n* = 5). **p < 0.01. **D** H&E staining of first mandibular molars from Lipo-PBS- and Lipo-Clo-treated mice at PN15.5 and PN21.5. Dotted lines indicate widened PDL space. **E** Quantification of PDL width at the furcation region at PN15.5 (*n* = 4) and PN21.5 (*n* = 3). *p < 0.05, **p < 0.01. **F** Immunofluorescence staining of periostin (red) in first mandibular molars at PN15.5 and PN21.5. Scale bars: 100 μm.

Our initial observations showed that macrophages persist throughout the course of root eruption, but their functional contribution to root development was unclear. To delineate their role, we depleted macrophages in vivo by administering clodronate liposomes (Lipo-Clo) or control liposomes (Lipo-PBS) at PN3.5, coinciding with the initiation of molar root morphogenesis (Fig. 2A). Immunofluorescence confirmed a marked reduction of macrophages in molars from the Lipo-Clo group, indicating effective local depletion (Fig. S2A, B). To exclude potential systemic confounders, we compared gross developmental parameters between groups. No significant differences were observed in body size, body weight, fur condition, spleen index, body length, and tibia length (Fig. S2C–G), indicating that macrophage depletion was locally restricted. In contrast, pronounced dental defects were observed. Macrophage-depleted mice exhibited shortened roots compared with controls, whereas crown morphology remained largely intact (Fig. 2B). Quantitative analysis confirmed a significant reduction in root length following macrophage depletion (Fig. 2C). Consistently, the impaired root elongation was accompanied by disorganized periodontal ligament at both PN15.5 and PN21.5 (Fig. 2D, E).

Given that periostin is a key extracellular matrix protein required for the integrity of the periodontium—including the PDL and surrounding alveolar bone [33, 34], we examined its expression from PN15.5 to PN21.5. Immunofluorescence staining results showed a significant reduction in periostin⁺ area within the PDL of Lipo-Clo mice (Fig. 2F), implying defects in PDL differentiation and maturation. Collectively, clodronate liposomes-mediated macrophage depletion significantly compromised root elongation and PDL maturation, underscoring a crucial role for macrophages in maintaining root homeostasis.

### Macrophage depletion leads to expansion of the Gli1^+^ mesenchymal progenitor pool

**Fig. 3:**
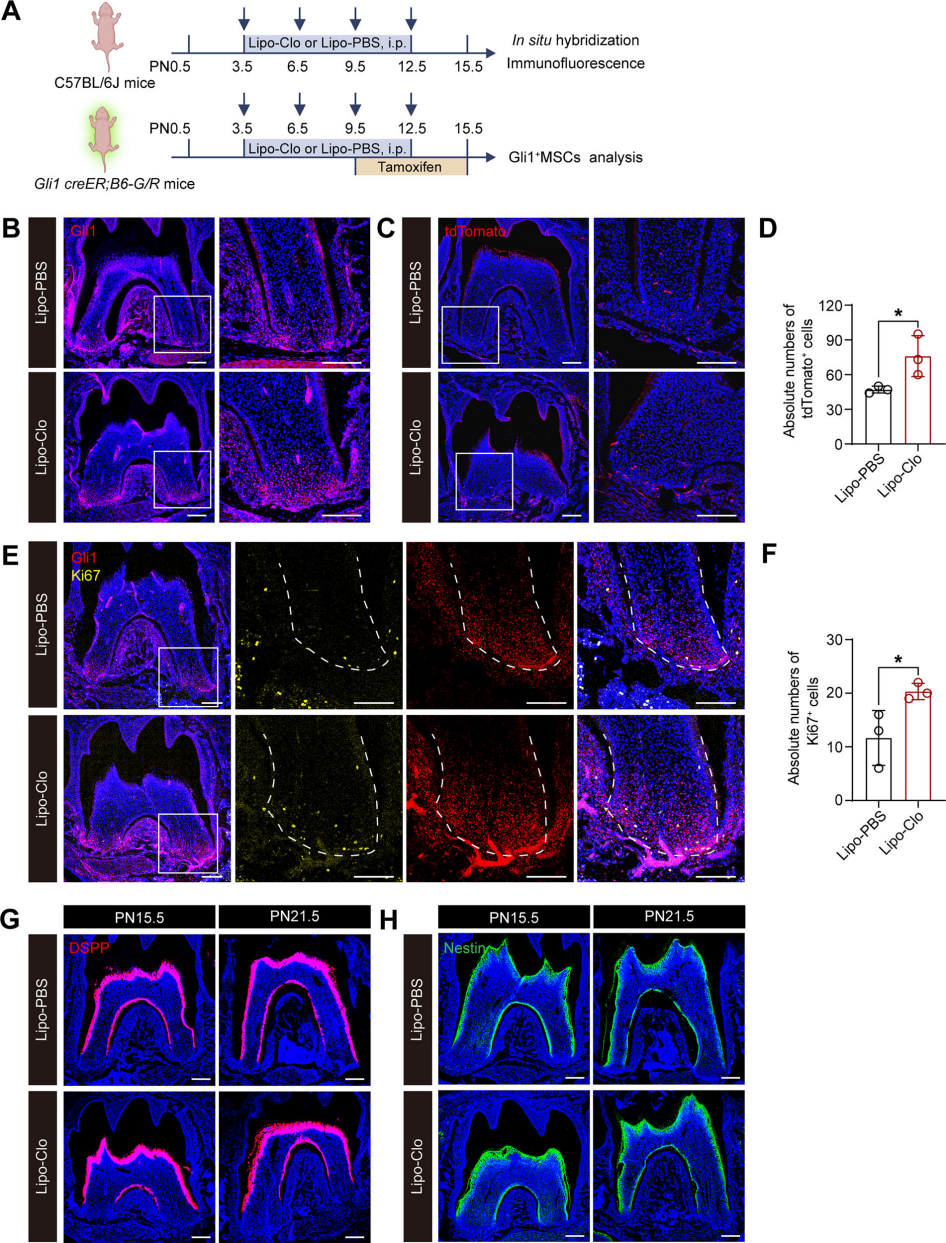
Macrophage depletion enhances proliferation of Gli1^+^ mesenchymal stem cells in developing molars. **A** Schematic of the experimental workflow: clodronate liposomes-mediated macrophage depletion combined with tamoxifen-induced tdTomato lineage tracing in Gli1⁺ cells (*Gli1-CreER;B6-G/R*). **B** RNAscope in situ hybridization for *Gli1* (red) in first mandibular molars of C57BL/6 J mice at PN15.5. **C** tdTomato⁺ Gli1^+^ cells (red) in first mandibular molars at PN15.5 in *Gli1-CreER;B6-G/R* mice. **D** Quantification of tdTomato⁺ cells in the apical root region (*n* = 3). ^*^p < 0.05. **E** RNAscope for *Gli1* (red) with Ki67 immunofluorescence (yellow) in C57BL/6 J molars at PN15.5. Dotted lines outline the root contour. **F** Quantification of Ki67⁺ cells in the apical region of Lipo-Clo versus control mice (*n* = 3). ^*^p < 0.05. **G** RNAscope for *Dspp* (red) in first mandibular molars of C57BL/6 J mice at PN15.5 and PN21.5. **H** Nestin immunofluorescence (green) in first mandibular molars at PN15.5 and PN21.5. Scale bars: 100 μm.

Extensive studies have established that Gli1⁺ cells act as multipotent tooth-root progenitors, contributing to odontoblasts, pulp, PDL, and adjacent alveolar bone [4, 35]. To define the cellular basis of the root defects associated with macrophage depletion, we performed inducible lineage tracing in *Gli1-CreER; B6-G/R* mice. Macrophages were depleted beginning at PN3.5, and Cre activity was induced with tamoxifen at PN9.5 (Fig. 3A). Transcriptome analysis via RNAscope in situ hybridization confirmed elevated *Gli1* expression in macrophage-depleted mice at P15.5 (Fig. 3B). Histologically, these mice showed an increased number of Gli1⁺ cells in the apical region of the root (Fig. 3C, D). The proliferative capacity of this population was further evidenced by a significant rise in Ki67⁺Gli1⁺ cells, supporting enhanced progenitor proliferation upon macrophage depletion (Fig. 3E, F).

The odontoblast marker DSPP is indispensable for dentin matrix assembly and mineralization [36]. However, no significant difference in *Dspp* expression was observed between the Lipo-Clo and Lipo-PBS groups at PN15.5 or PN21.5 by in situ hybridization (Fig. 3G). Consistently, immunofluorescence staining for Nestin (a marker of odontoblast differentiation) showed comparable localization and signal intensity in odontoblasts (Fig. 3H). The comparable expression of *Dspp* and Nestin in MSCs, but not periostin, may reflect distinct regulatory mechanisms governing lineage-specific differentiation.

### Downregulation of osteogenic differentiation-related genes in dental mesenchymal cells upon macrophage depletion

**Fig. 4:**
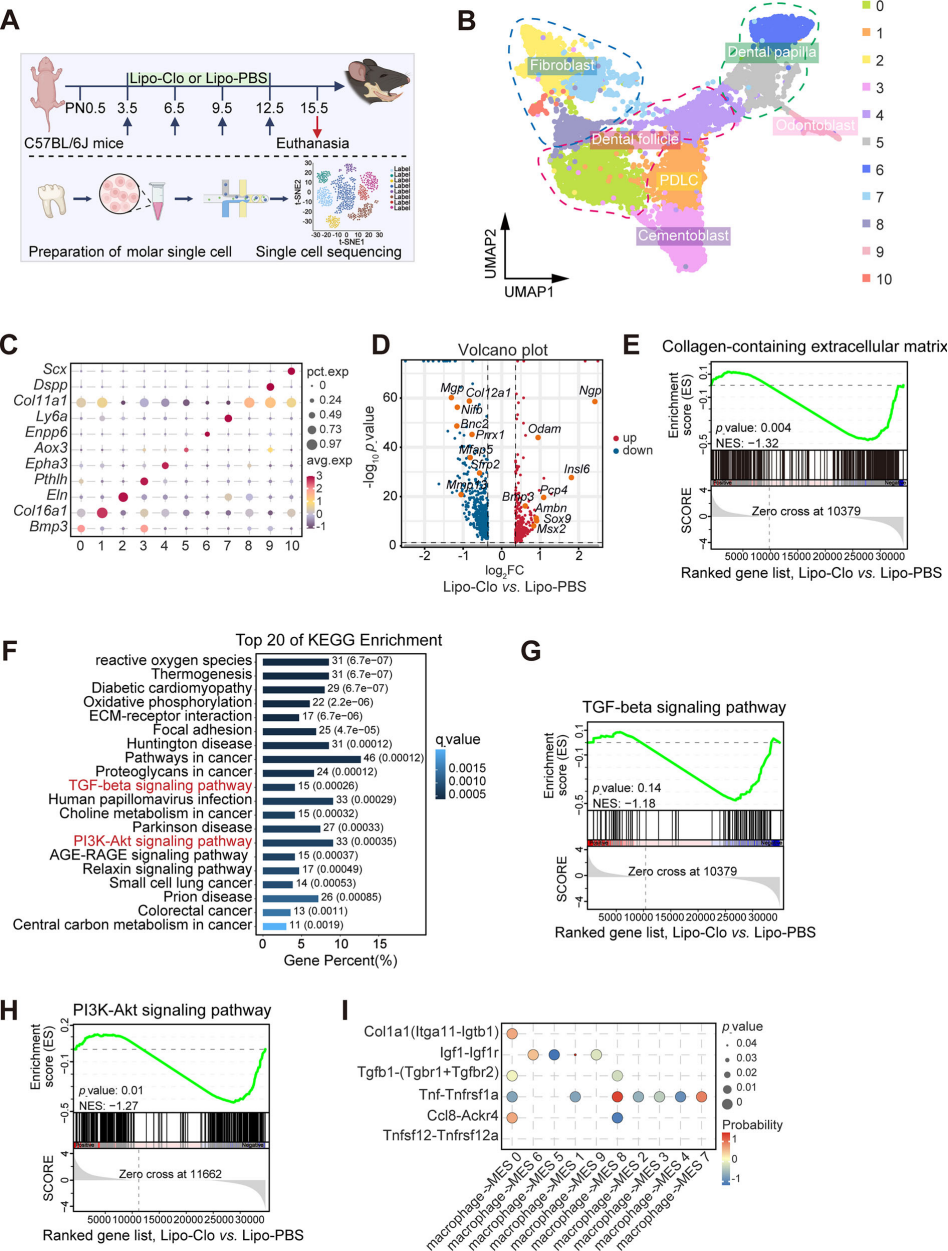
Macrophage depletion downregulates osteogenic programs in dental mesenchyme. **A** Schematic of the scRNA-seq workflow for PN15.5 mandibular first molars and peri-molar tissues after Lipo-Clo-mediated macrophage depletion and Lipo-PBS controls. **B** UMAP visualization of dental mesenchymal cells identified by scRNA-seq. PDLCs: periodontal ligament cells. **C** Dot plot showing cluster-defining marker genes across mesenchymal subclusters. **D** Volcano plot of differentially expressed genes (DEGs) in dental mesenchyme (Lipo-Clo versus Lipo-PBS). **E** GSEA analysis indicating downregulation of osteogenic programs in Lipo-Clo mice relative to controls. **F** Top 20 KEGG pathways enriched in DEGs. **G**, **H** GSEA showing downregulation of TGF-β and PI3K-AKT signaling pathways in dental mesenchyme following macrophage depletion. **I** Predicted cellular crosstalk between macrophages and dental mesenchyme.

To define the cellular mechanisms and functional contributions of macrophages during root development, we performed single-cell RNA sequencing of mandibular first molars and surrounding tissues at PN15.5 following macrophage depletion (Fig. 4A). Transcriptomic profiling revealed a complex tissue landscape comprising endothelial cells, dental mesenchymal cells, macrophages, lymphocytes, and neutrophils (Fig. S3A). Cell identities were validated by canonical markers, including *Col1a1* and *Eln* (mesenchyme), *Cdh5* (endothelium), *Hba-a2/Hbb-bt* (erythrocytes), *Top2a* (cycling cells), *Apoe* (macrophages), *Rgs5* (pericytes), *Cd74* (lymphocytes), *Krt14/Amelx* (epithelium), and *S100a8* (neutrophils) (Fig. S3B, C) [37].

Subclustering of dental mesenchyme, annotated by known mesenchymal markers and dimensionality reduction, gave rise to 10 transcriptionally distinct subclusters, underscoring substantial heterogeneity (Fig. 4B, C). Differential expression analysis identified 1,050 differentially expressed genes (718 upregulated; 332 downregulated). Notably, downregulated genes included *Prrx1*, *Mfap5*, *Sfrp2*, and others implicated in MSC osteogenic differentiation (Fig. 4D). Gene set enrichment analysis (GSEA) further demonstrated downregulation of collagen-containing extracellular matrix signatures in macrophage-depleted sample (Fig. 4E). KEGG pathway analysis revealed downregulation of TGF-β and PI3K-AKT signaling, alongside pathways related to ECM-receptor interaction and focal adhesion (Fig. 4F–H). Notably, TGF-β and PI3K-AKT pathways are known to be critically required for MSC osteogenic differentiation through crosstalk with Wnt and MAPK signaling, upregulating alkaline phosphatase (ALP) and osteocalcin (OCN) expression to direct osteoblast lineage commitment [38, 39].

To interrogate intercellular communications, we applied CellChat (package32) to map ligand–receptor networks between macrophages and spatially distinct mesenchymal domains. Macrophages emerged as a major source of growth-factor ligands to multiple mesenchymal subpopulations (Fig. 4I). Analysis of an independent public scRNA-seq dataset (GSE189381) validated these patterns, highlighting macrophages as key emitters of TGF-β signals that influence the fate of multiple MSC subclusters (Fig. S4A, B).

Notably, our scRNA-seq data revealed four dental macrophage subsets in PN15.5 molars: CCL2^hi^ (chemokine-enriched immunomodulatory), Col1a1^hi^ (ECM-remodeling), Lgals3^hi^ (antigen-presenting), and ACP5^hi^ (osteoclast-primed) populations (Fig. S4C–F). Depletion of macrophages leads to a reduction in Col1a1^hi^ TGF-β^hi^ subset (Fig. S4E), which could disrupt the ECM remodeling process, suggesting specialized roles wherein distinct subsets may spatially deliver TGF-β or other ligands to MSC niches.

### Macrophage-derived signals enhance osteogenic differentiation of mesenchymal stem cells

**Fig. 5:**
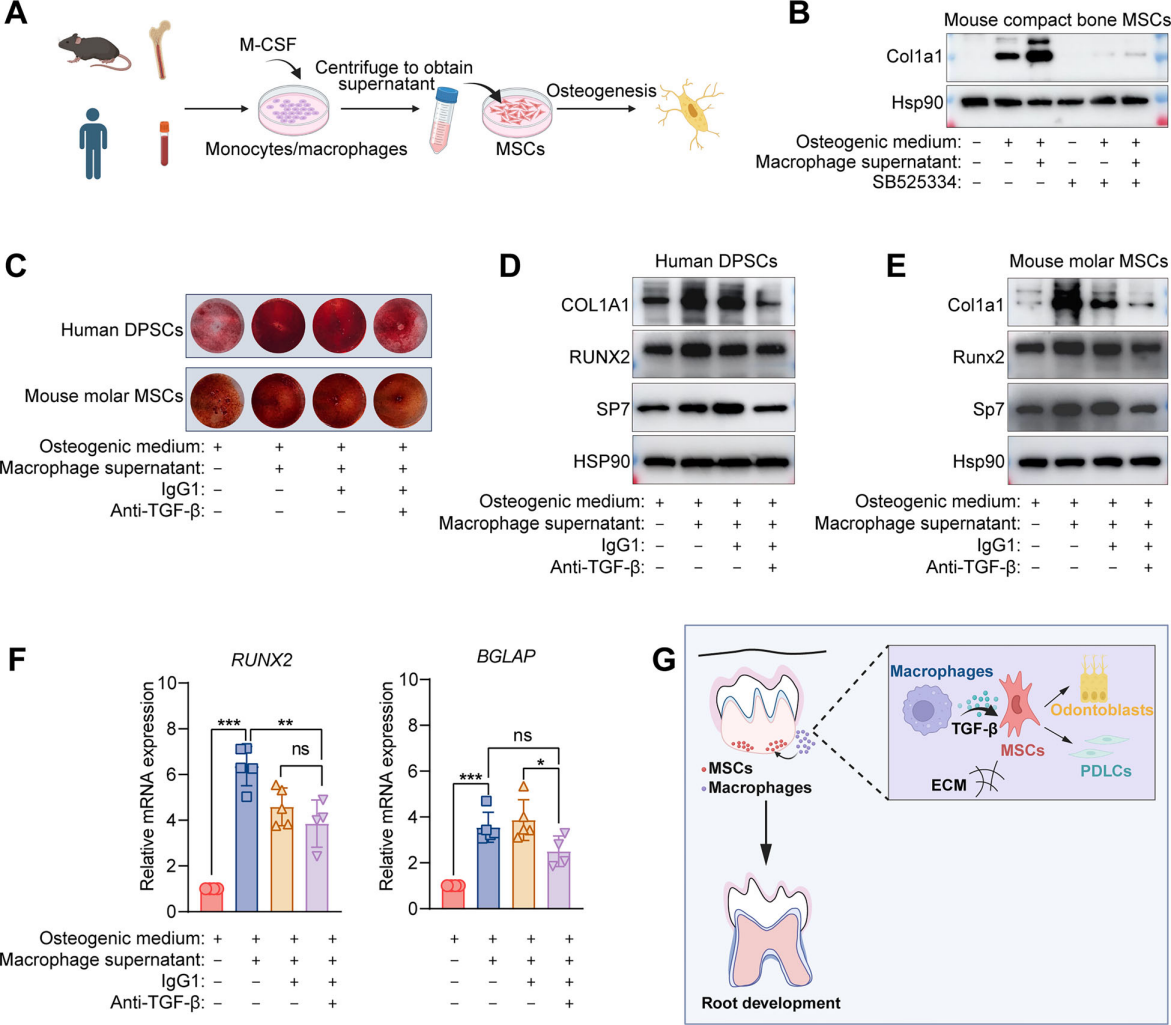
Monocyte/macrophage regulation of MSC osteogenic differentiation. **A** Schematic of in vitro assays assessing macrophage effects on MSC osteogenic differentiation. **B** Macrophage-derived supernatants promote osteogenic differentiation of compact bone-derived MSCs. **C** ARS staining of Human DPSCs and mouse molar MSCs after TGF-β neutralization. **D** Representative Western blot image of COL1A1, RUNX2 and SP7 levels in Human DPSCs. **E** Representative Western blot image of Col1a1, Runx2 and Sp7 levels in mouse molar MSCs. **F** mRNA levels for *RUNX2* and *BGLAP* of Human DPSC after TGF-β neutralization. *p < 0.05, **p < 0.01, ***p < 0.001; ns, not significant. **G** Model summarizing the regulatory role of macrophages in MSC osteogenic differentiation via secretion of TGF-β ligands, contributing to proper root development. PDLCs: periodontal ligament cells.

Next, we isolated MSCs from mouse compact bone and expanded them in vitro. P3 MSCs were characterized by flow cytometry and exhibited the classic MSC surface marker profile (Fig. S5A). To evaluate the regulatory effects of macrophages on MSCs, we cultured MSCs in macrophage-conditioned medium (Fig. 5A). Notably, conditioned medium derived from bone marrow (BM) monocytes/macrophages exerted a stronger pro-osteogenic effect, as evidenced by significantly elevated Col1a1 protein expression. In contrast, TGF-β receptor inhibition markedly reduced Col1a1 levels (Fig. 5B). Given that MSCs are receiving tremendous interest in the field of developmental biology [40, 41]. To avoid MSC autocrine signaling and further elucidate the specific contribution of macrophage-derived paracrine factors, we selectively neutralized TGF-β activity in macrophage-conditioned medium using a TGF-β-neutralizing antibody. Ontogenetically, compact bone-derived MSCs (mesodermal) and DPSCs (ectodermal) diverge in embryonic origin. Therefore, we isolated Human dental pulp stem cells and mouse molar MSCs and expanded them in vitro (Fig. S5B, C). The conditioned medium derived from human PBMC-derived macrophages or mouse bone marrow-derived M2 macrophages supports the above finding. Alizarin red (ARS) staining proved that both conditioned media promoted the osteogenic differentiation of MSCs (Fig. 5C). In addition, the protein levels of conserved osteogenic markers (human *SP7*/mouse *Sp7*, human *RUNX2*/mouse *Runx2*, and human *COL1A1*/mouse *Col1a1*) were significantly upregulated (Fig. 5D, E). Consistently, the mRNA expression of osteogenic genes (*RUNX2* and *BGLAP*) was also markedly increased in human DPSC (Fig. 5F).

Taken together, our study identifies the functional macrophages critical for proper root morphogenesis, whose depletion causes impaired root elongation and disorganized PDL architecture. Mechanistically, these macrophages potentiate MSC osteogenic differentiation through TGF-β secretion, ultimately orchestrating root developmental patterning (Fig. 5G).

## Discussion

The immune microenvironment is increasingly recognized as a key regulator of organogenesis. Beyond their classical roles in inflammation and host defense, immune cells actively participate in organ formation, cell-fate decisions, and tissue homeostasis [42, 43]. Among immune cells, macrophages are particularly versatile, owing to their diverse subtypes, secretome, and dynamic intercellular interactions. They perform complex regulatory functions in various tissues that go far beyond their classical role as phagocytic “scavengers” [44, 45]. Here, we show that macrophages are pivotal for tooth root development. Depletion of macrophages in the root developmental niche led to defective differentiation of roots and periodontal ligaments. Mechanistically, single-cell RNA sequencing and in vitro co-culture assays indicated that macrophages shape the root niche via TGF-β signaling, which governs dental mesenchymal stem cell lineage commitment during molar root development.

The root developmental microenvironment hosts a spectrum of heterogeneous macrophages. F4/80⁺CD68⁺ macrophages progressively expanded in abundance and spatial distribution throughout the dental pulp and PDL during root formation, displaying an M2-like phenotype (CD206⁺). Meanwhile, CD68⁺F4/80⁻ osteoclasts in the dental follicle and alveolar bone mediated bone remodeling to facilitate tooth eruption. Both macrophages, though functionally distinct, collaborate as myeloid-lineage regulators of skeletal morphogenesis [24, 46]. Single-cell profiling further confirmed the heterogeneity in macrophages and revealed four distinct dental macrophage subsets in PN15.5 molars, including CCL2^hi^ (chemokine-enriched immunomodulatory), Col1a1^hi^ (ECM-remodeling), Lgals3^hi^ (antigen-presenting), and ACP5^hi^ (osteoclast-primed) populations. Notably, depletion of macrophages leads to a reduction in Col1a1^hi^ subset, thereby compromising the ECM remodeling process, suggesting subset-specific macrophage regulation of MSC lineage commitment.

Tooth root development is a tightly regulated process requiring coordinated actions of multiple cell types and precise molecular cues [2, 47]. Central to this program are Gli1⁺ MSCs [35, 48], which serve as key dental mesenchymal progenitors that generate odontoblasts and dental follicle-derived lineages essential for root formation. These cells also respond to multiple developmental cues such as Sonic Hedgehog, TGF-β, and BMP signals, orchestrating root morphogenesis, matrix mineralization, and functional maturation [27, 49, 50]. Using tdTomato to trace the Gli1⁺ lineage cells of developing molars, we observed a significant expansion of the Gli1⁺ MSC pool, yet impaired differentiation into functional periodontal cells (marked by reduced periostin expression) following macrophage depletion. Surprisingly, no significant differences in the expression levels or spatial distribution of classic markers *Dspp* and Nestin between macrophage-depleted and control groups. This suggests that macrophage-MSC crosstalk selectively regulates lineage-specific differentiation rather than broadly disrupting odontogenic programs.

The expansion of Gli1⁺ MSCs in macrophage-deficient molars indicates a disruption of the normal balance between progenitor maintenance and lineage commitment. Mechanistically, macrophages likely orchestrate Gli1⁺ MSC fate decisions via dual modalities: (i) secreting inhibitory cues that restrain excessive stem-cell proliferation to maintain an appropriate progenitor pool; and (ii) activating differentiation programs via TGF-β and related pathways that guide progenitors toward functional odontoblasts. Loss of macrophages disrupts this dual control, driving aberrant Gli1⁺ MSC proliferation coupled with differentiation arrest, collectively impairing root morphogenesis. Consistently, a recent study demonstrated a critical role of macrophages in antler generation in male sika deer, and even female deer could generate antlers when autologous macrophages were locally injected [51]. These conserved findings underscore macrophages as critical regulators of mesenchymal fate determination, though their context-dependent molecular effectors require further delineation.

Historically, the absence of reliable in vitro differentiation models and in vivo lineage-tracing tools has limited detailed interrogation of root microenvironmental cues. Single-cell transcriptomics now enables high-resolution mapping of intercellular communication during organogenesis. Using scRNA-seq, we found that macrophage depletion significantly downregulated osteogenic/odontogenic genes in dental mesenchyme (e.g., *Prrx1, Sfrp2, Mgp*), a transcriptional program underlying the shortened roots and defective mineralization observed. In addition, we detected reduced cell-adhesion signaling and decreased expression of TGF-β pathway components in Lipo-Clo-treated mice. Together, these data delineate a multifaceted signaling network in which macrophages coordinate multiple pathways to fine-tune tooth root morphogenesis. In vitro assays further validated that macrophage-conditioned medium significantly promoted osteogenic differentiation of MSCs. Critically, TGF-β signaling blockade suppressed the pro-osteogenic response, establishing TGF-β as a crucial regulator of macrophage-dependent MSC differentiation.

Collectively, our findings highlight macrophage-derived TGF-β as a critical driver of immune-mesenchymal crosstalk during tooth root development, revealing the essential role of macrophages in orchestrating organ development.

## Materials and methods

### Mice

C57BL/6 J mice, aged 8–10 weeks, were purchased from Slack Laboratory Animal Technology Co., Ltd. (Shanghai, China). *Gli1-CreER* and *B6-G/R* transgenic mice were purchased from GemPharmatech (Nanjing, China) and used for lineage tracing experiments. Mice were maintained in specific pathogen-free conditions and euthanized using CO_2_ inhalation followed by cervical dislocation. Both sexes mice were included in the study.

### Animal procedures

*Gli1-CreER* male mice were crossed with *B6/JGpt-H11*^*em1Cin(CAG-loxP-ZsGreen-Stop-loxP-tdTomato)*^*/Gpt (B6-G/R)* female mice. Mice homozygous for this *ZsGreen/tdTomato* mutation are viable and fertile. The green fluorescent protein ZsGreen binds to the red fluorescent protein tdTomato and is knocked into the H11 gene locus of mouse origin. These mice possess *loxP* sites on either side of a membrane-targeted *ZsGreen* cassette and express strong green fluorescence in all tissues and cell types examined. When bred to Cre recombinase expressing mice, the resulting offspring have the *ZsGreen* cassette deleted in the Cre expressing tissue(s), activating the expression of tdTomato and emitting dazzling red fluorescence. Due to the tissue-specific expression of Cre, mice follow the expression time and location of Cre to turn on red fluorescence.

### Clodronate liposomes and tamoxifen administration

C57BL/6 J and *Gli1-CreER; B6-G/R* mice received intraperitoneal injections of clodronate liposomes (YiSheng, China) at a dosage of 40 μg/g every third day. Control animals received an equivalent volume of vehicle liposomes (Lipo-PBS). Tamoxifen (Sigma, T5648) was dissolved in corn oil (Sigma, C8267) at a concentration of 20 mg/mL. Cre recombination was induced with tamoxifen injected intraperitoneally in mice at a dose of 75 μg/g at PN9.5. The allocation of test animals used in our study was random. There was no targeted selection of individual animals for specific treatments. No statistical methods were used to pre-determine sample sizes; however, the sizes employed are in line with previously published studies in the field.

### Imaging mass cytometry staining

Tooth samples were fixed in 4% paraformaldehyde (PFA) in PBS for 2 days at 4 °C, washed 3 times with PBS, and decalcified in a 10% EDTA pH 7.2 solution at 4 °C for 2–3 weeks, depending on the age of the samples. After washing with PBS, samples were dehydrated in 30% sucrose in PBS at 4 °C for 2 days and processed for frozen sections. Sections were fixed with 4% PFA for 30 min at 4 °C and then washed the slides in Maxpar PBS for 5 min at room temperature. Frozen sections were blocked with the prepared 3% BSA blocking solution in Maxpar PBS for 45 min at room temperature. Incubate the slides with the antibody cocktail overnight at 4 °C in a hydration chamber (antibody details see Supplementary Table). After being washed three times with Maxpar PBS, sections were stained with the prepared Intercalator-Ir in Maxpar PBS for 30 min at room temperature. Subsequently, sections were washed in double-distilled water for 5 min and air-dried for 20 min at room temperature. Finally, tissue sections were analyzed by imaging mass cytometry, which couples laser ablation techniques and CyTOF mass spectrometry.

### Histological analysis

After fixation and decalcification, mandibles were cryoprotected in graded sucrose solutions and embedded in OCT compound (Tissue-Tek, Sakura). Cryosections of 8 μm thickness were prepared using a Leica CM3050S cryostat. Hematoxylin and eosin (H&E) staining was performed following standard protocols.

### TRAP staining

TRAP staining was performed using the TRAP/ALP staining kit (Wako, 294-67001) to detect osteoclasts, according to the manufacturer’s protocol. In brief, mouse mandible sections were incubated with 0.5 mL TRAP staining solution for 30 min at room temperature in a humidified chamber. Sections were rinsed three times with distilled water, followed by application of 0.5 mL nuclear staining solution.

### Micro-CT analysis

Mouse mandibles were harvested and fixed overnight in 4% paraformaldehyde, followed by decalcification in 10% EDTA (pH 7.2) for 3 weeks. After fixation, mandibles were dried, wrapped in parafilm, and scanned using a SkyScan 1176 Micro-CT imaging system (Bruker, Germany). Scanning parameters included: 50 kV voltage, 455 μA current, 265 ms exposure, 18 μm resolution, and 0.8° rotation step. Images were reconstructed using NRecon, visualized with CTvox, and analyzed using CTAn software. The first molar alveolar bone was selected as the region of interest (ROI), and identical analysis parameters were applied across all samples.

### Immunofluorescence and in situ hybridization

Samples were prepared as above. Following decalcification and PBS washing, samples were soaked in PBS containing 30% sucrose at 4 °C for 2 days. Tissue was cut at 8 μm thickness using a Cryostat Leica CM3050S with high-profile microtome blades and cryofilm (Section Laboratory). Before staining with antibodies, cryosections were incubated with blocking buffer containing 3% BSA and 0.3% Triton™ X-100 in PBS for 1 h at room temperature. Sections were washed twice with PBS for 5 min, then incubated overnight at 4 °C with primary antibodies diluted in blocking buffer. After washing three times with PBS, sections were incubated with Alexa Fluor-conjugated secondary antibodies (Thermo Fisher), counterstained with DAPI (Abcam, C1002), and imaged.

In situ hybridization was performed using RNAscope Multiplex Fluorescent Reagent Kit (Advanced Cell Diagnostics, 323100) and RNA-Protein Co-Detection Ancillary Kit (Advanced Cell Diagnostics, 323180), according to the manufacturer’s instructions. In brief, tissues were fixed in 4% PFA overnight at 4 °C and subsequently cryosectioned into 8-μm-thick slices. Following dehydration with graded ethanol, antigen retrieval was performed on the sections at 98–102 °C for 5 min, then treated with protease for 10 min at room temperature. If RNA-Protein Co-Detection were required, sections were incubated overnight at 4 °C with primary antibodies (Rabbit anti-Ki67, Abcam, ab15580). Probes were then hybridized for 2 h at 40 °C. Subsequently, RNAscope amplification reagents (provided in RNAscope Multiplex Fluorescent Reagent Kit v2) were applied according to the manufacturer’s protocol. Finally, signal detection was performed with TSA Plus Cyanine 3.

### Cell culture

All cell culture experiments were conducted under sterile conditions. Mouse bone marrow-derived macrophages (BMDMs) were isolated from C57BL/6 J mice, while human PBMC-derived macrophages were differentiated from peripheral blood mononuclear cells of healthy donors. Mouse MSCs and human MSCs were obtained from C57BL/6 J mice and healthy donors, respectively. All cells were maintained in DMEM supplemented with 10% fetal bovine serum (FBS), 1× penicillin–streptomycin-amphotericin B antibiotic/antimycotic solution, at 37 °C under 5% CO₂.

### Differentiation of monocyte-derived macrophage

PBMCs were isolated from whole blood collected from healthy adult donors. Briefly, whole blood was diluted 1:1 with PBS and carefully layered over Lymphoprep™ (StemCell Technologies) density gradient media. After centrifugation at 800 × *g* for 20 min at room temperature without brake off, the PBMC fraction was collected without disturbing the plasma: Lymphoprep™ interface. PBMCs were then washed with PBS at 300 × *g* for 10 min. CD14^+^ monocytes were magnetically sorted from PBMCs using EasySep™ Human CD14 Positive Selection Kit II (StemCell Technologies) and cultured in DMEM supplemented with 10% FBS and 20 ng/mL macrophage colony-stimulating factor (M-CSF, GenScript, Z02914-1). During the differentiation of macrophages, half of the media was changed at day 3. At day 7, the differentiated macrophages were harvested.

### BMDM differentiation

BMDMs were differentiated from bone marrow hematopoietic progenitors under sterile conditions. In brief, mouse femurs and tibiae were dissected, and the bone marrow was extruded by flushing the medullary cavity with cold PBS through a 25-gauge needle. The cell suspension was filtered through a 70-μm strainer before culture. The isolated cells were cultured in DMEM supplemented with 10% FBS and 20 ng/mL macrophage colony-stimulating factor (M-CSF, GenScript, Z02930-50). During the propagation and differentiation of BMDMs, half of the media was changed at day 3. At day 5, 20 ng/mL recombinant murine IL-4 (NOVUS, NBP2-35131) was optionally added to induce M2 macrophage polarization. At day 7, macrophages were treated with either 40 μg/mL anti-TGF-β neutralizing antibody or IgG isotype control (Selleck, A2113).

To harvest PBMC or macrophage-conditioned medium, culture supernatants were collected and centrifuged at 1000 × *g* for 10 min to remove cellular debris. For functional assays, BMDMs/PBMC-derived macrophage conditioned medium was mixed 1:1 (v/v) with either DMEM medium (as a control) or osteogenic differentiation medium prior to treatment.

### Isolation and culture of MSCs from mouse compact bone

Primary MSCs were isolated from 2-week-old C57BL/6 J mice euthanized via cervical dislocation following institutional animal care guidelines. Cut the femurs and tibias from which the bone marrow has been washed out into 1-3 mm^3^ fragments; Digest the bone fragments with α-MEM supplemented with 2% FBS and 1.0 mg/mL collagenase type II (Gibco) at 37 °C with constant agitation (200 rpm) for 1 h. Stop digestion when the bone fragments become loose. After centrifugation at 300 × *g* for 5 min, the collagenase-containing supernatant was carefully aspirated. The digested bone fragments were subsequently seeded in the culture dish, and an appropriate amount of fresh α-MEM (containing 10% FBS) was added to submerge the bone fragments; the culture dish was placed in an incubator at 37 °C and 5% CO2. The culture medium was routinely replaced every 3 days. When the confluence reaches 80–90%, digest with trypsin-EDTA solution (0.25%:0.02%) and passage at a ratio of 1:4.

### Isolation and culture of MSCs from first mandibular molars

Dental pulp mesenchymal cells (DPCs) were isolated from first mandibular molars harvested from C57BL/6 J mice aged PN7.5. The tissues were carefully minced into small fragments using sterile surgical blades and then cultured in α-MEM medium supplemented with 10% fetal bovine serum at 37 °C in a 5% CO_2_ incubator. The culture medium was routinely replaced every 3 days.

For MSCs derived from mouse mandibular first molars and compact bone, primary cells were expanded to passage 3 (P3) prior to characterization. P3 MSCs were detached with 0.25% trypsin/EDTA and stained with antibodies against CD44, CD29, Sca-1, CD45, CD31 and CD34 for flow cytometric characterization (antibody details see Supplementary Table).

### Isolation and culture of primary human dental pulp cells

Primary dental pulp stem cells (DPSCs) were isolated from healthy third molars extracted from adult donors. The extracted teeth were washed three times in sterile PBS and then tapped with a sterile surgical mallet to expose the pulp chamber. The pulp tissue was gently dissected from the crown and root, followed by enzymatic digestion in 3 mg/mL Collagenase I (Gibco) and 4 mg/mL Dispase Ⅱ (Roche) at 37 °C for 1 h. Cells were filtered (70 µm strainer) to obtain single-cell suspensions. The isolated cells were cultured in DMEM (containing 10% FBS). Place the culture dish in an incubator at 37 °C and 5% CO_2_. Change the culture medium every 3 days; When the confluence reaches 80–90%, digest with trypsin-EDTA solution (0.25%:0.02%) and passage at a ratio of 1:4. P3 MSCs were detached with 0.25% trypsin/EDTA and stained with antibodies against CD90, CD29, CD146, CD73, CD105, CD45, CD31 and CD34 for flow cytometric characterization.

### Osteogenic differentiation

1 × 10^5^ MSCs/well were seeded into a 12-well plate. To induce osteogenic differentiation, MSCs were cultured with osteogenic differentiation medium, which consisted of α-MEM medium supplemented with 50 μg/mL ascorbic acid (Sigma, A8960), 5 mM β-glycerophosphate (Sigma, G9422), and 100 nM dexamethasone (Sigma, D4902), with conditioned medium added at a 1:1 ratio (v/v). The compact bone-derived MSCs culture medium was supplemented with 2 μM ALK5 inhibitor (SB525334, Selleck, S1476) to specifically inhibit the TGF-β signaling pathway.

### ARS staining

Mineralized bone nodules were stained with 2% Alizarin Red S solution (Beyotime, C0138) for 15 min at room temperature. Subsequently, the samples were rinsed with PBS to eliminate unbound stain.

### RNA isolation and RT-qPCR analysis

RNA was extracted from primary mouse MSC or human DPSCs using FastPure Cell/Tissue Total RNA Isolation Kit V2 (Vazyme, Nanjing, China), and cDNA was synthesized using PrimeScript™ RT Master Mix (Takara, Dalian, China). Real-time PCR was performed using SYBR qPCR Master Mix (Vazyme, Nanjing, China) according to the manufacturer’s guidelines on QuantStudio 6 Flex (Applied Biosystems, Foster City, CA). The following primers were used for qPCR: *Runx2* (5′- CACTGGCGCTGCAACAAGA-3′, 3′- CATTCCGGAGCTCAGCAGAATAA-5′), *Bglap* (5′-AGCAAAGGTGCAGCCTTTGT-3′, 3′-GCGCCTGGTCTCTTCACT-5′), *β-actin* (5′-TGGCACCCAGCACAATGAA-3′, 3′-CTAAGTCATAGTCCGCCTAGAAGCA-5′). Relative expression was calculated as RQ = 2^–ΔΔCt^.

### Western blot

The MSCs cultured in vitro were washed twice with cold PBS and lysed in RIPA buffer containing a complete protease inhibitor cocktail on ice for 30 min. Cell lysates were then centrifuged at 12,000 rpm for 10 min at 4 °C to obtain the supernatant. Western blot was performed per standard protocol. The following primary antibodies were incubated: anti-Col1a1 (Cell Signaling Technology, 72026S), anti-Runx2 (Cell Signaling Technology, 12256S) and anti-Sp7(Abcam, ab209484) at 4 °C overnight. After incubation with the HRP-conjugated secondary antibodies (Cell Signaling Technology, 7074S) for 1 h at room temperature, the signal was visualized with ECL reagents (FDbio) and detected by a chemiluminescence imaging system (Cytiva, Amersham ImageQuant™ 800).

### Flow cytometry

For single-cell preparation, first mandibular molars and surrounding tissues from 8 to 10-week-old C57BL/6 J mice were enzymatically digested using 4 mg/mL Dispase Ⅱ (Roche) and 2 mg/mL Collagenase I (Gibco) (37 °C, 30 min; Eppendorf thermomixer). The liberated cells were filtered (40 μm nylon mesh) and collected by low-speed centrifugation (400 × *g*, 5 min). Single-cell suspensions from molar tissues were incubated with antibody cocktail in PBS with 2% FBS, for 30 min at 4 °C in the dark. Washing steps with PBS (containing 10% FBS) were repeated three times. Anti-mouse antibody information used for staining is described in the Supplementary Table. Cell acquisition was performed on an LSRII (BD Biosciences). Data were analyzed with FlowJo software (Tree Star).

### Cell isolation and single-cell RNA sequencing

To obtain single-cell transcriptomes, first mandibular molars and their surrounding tissues at PN15.5 were digested with 4 mg/mL Dispase (Roche) and 2 mg/mL Collagenase I (Gibco) at 37 °C for 30 min on a thermomixer (Eppendorf). The resulting cell suspension was filtered through a 40-μm strainer (Corning, 352350) and centrifuged at 400 × *g* for 5 min. Cell viability and counts were assessed using an automated cell counter. Samples were then used for single-cell RNA sequencing with the 10x Genomics system m. Library sequencing was performed on the Illumina NovaSeq X Plus by Gene Denovo Biotechnology Co., Ltd (Guangzhou, China). Bioinformatics analyses were performed using Omicsmart (http://www.omicsmart.com).

### Statistical analysis

No animals or samples were excluded from the analysis. For all animal experiments, investigators were blinded to the group allocation during the data collection and analysis. Statistical analysis was performed using GraphPad Prism 9. Data are presented as mean ± SEM. Two-group comparisons used Student’s *t* test if F-test indicated equal variances. For multiple group comparisons, one-way ANOVA followed by Tukey’s post hoc test was performed when appropriate. Experiments were repeated at least twice. Significance thresholds were set at *p < 0.05, **p < 0.01, ***p < 0.001; ns, not significant.

## Supplementary information

Supplementary Information

Supplementary Table1

Original Data

## Data Availability

Single-cell RNA-seq data generated in this study are available through the GEO database under accession code GSE320526. The publicly available scRNA-seq data of Supplementary Figure 4A and B were obtained from the datasets deposited in the GEO database GSE189381 (Jing J et al., *Nat Commun*, 2022, PMID: 35974052) and reanalyzed by RStudio (version 2023.12.0.369) and R (version 4.3.2).
