# Supplementary material for: Depletion of macrophages during early postnatal development leads to disrupted tooth root development and altered Gli1⁺ MSC trajectory
Source: Cell Death Dis. 2026 Apr 26;17(1):555. doi: 10.1038/s41419-026-08753-7 (PMC13247052; doi:10.1038/s41419-026-08753-7)
Supplement: Supplementary file 1 — Supplementary Information [file 41419_2026_8753_MOESM1_ESM.pdf]

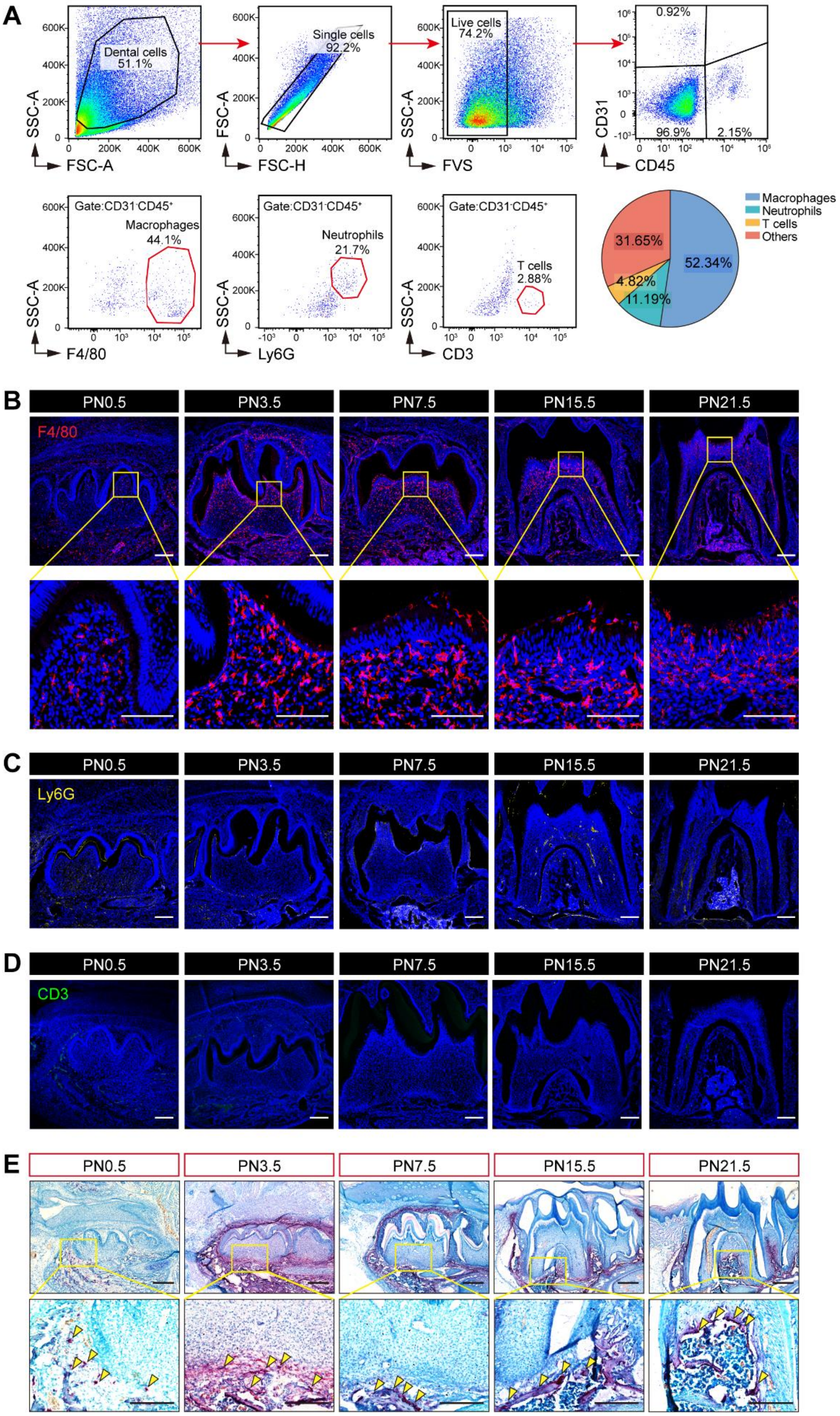

Fig. S1 Spatial distribution of immune cells during molar root development.

A, Representative flow cytograms and immune cell proportions (pie chart) in mouse first mandibular molars.

B, Immunofluorescence staining of F4/80$^+$ macrophages in first mandibular molars from PN0.5 to PN21.5. Yellow areas highlight macrophage localization within the odontoblast layer.

C, Immunofluorescence staining of Ly6G$^+$ neutrophils in first mandibular molars from PN0.5 to PN21.5.

D, Immunofluorescence staining of CD3$^+$ T cells in first mandibular molars from PN0.5 to PN21.5.

E, TRAP staining reveals the spatial distribution of osteoclasts during molar development. Yellow arrows indicate TRAP$^+$ cells. Scale bars: 100 μm.

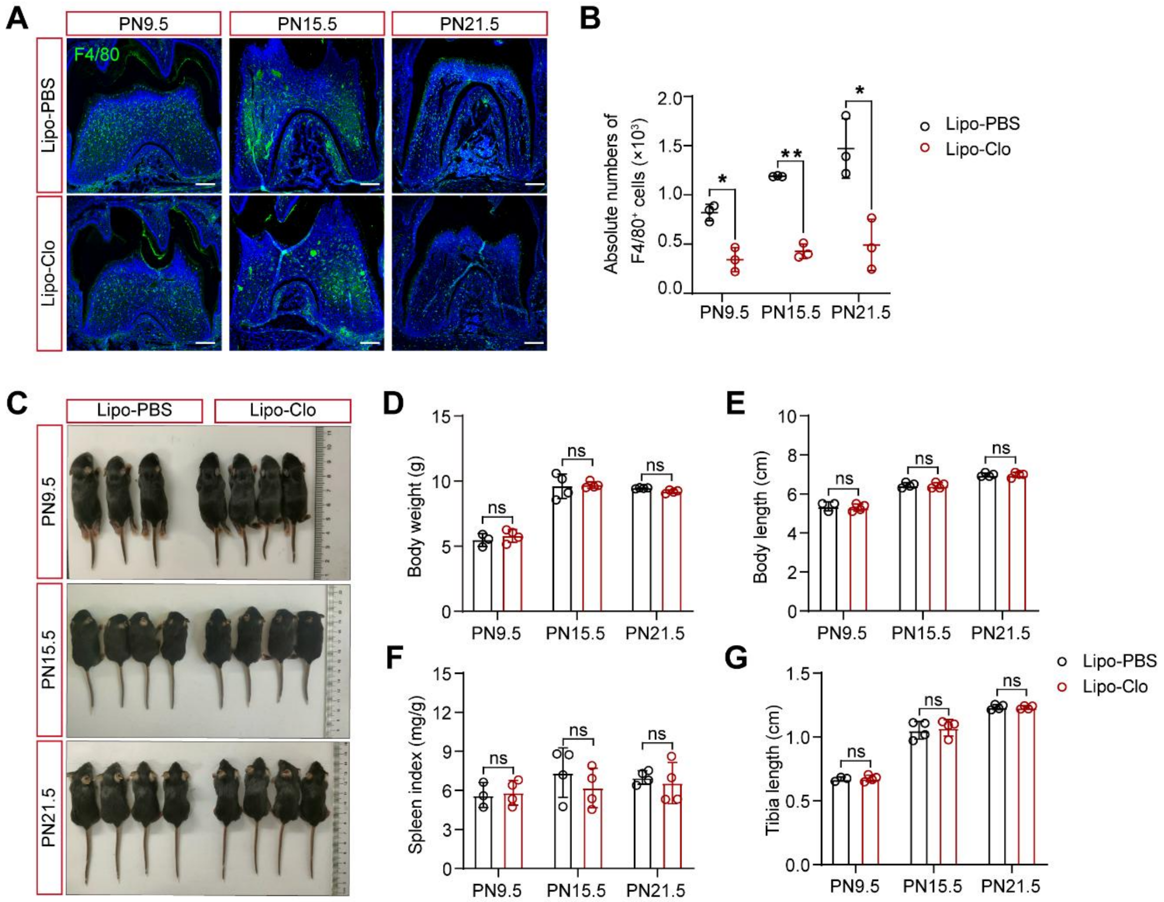

Fig. S2 Clodronate liposomes efficiently deplete macrophages *in vivo*.

A, Immunofluorescence staining of F4/80$^+$ macrophages in first mandibular molars of Lipo-Clo- and Lipo-PBS-treated mice at PN9.5, PN15.5, and PN21.5.

B, Quantification of F4/80$^+$ macrophages at the indicated developmental stages. *$p < 0.05$, **$p < 0.01$.

C, Photographs of mice at the indicated developmental stages.

D, Quantification of body weight at the indicated developmental stages.

E: Quantification of body length at the indicated developmental stages.

F: Quantification of spleen index at the indicated developmental stages.

G: Quantification of tibia length at the indicated developmental stages. ns, not significant. Scale bars: 100 μm.

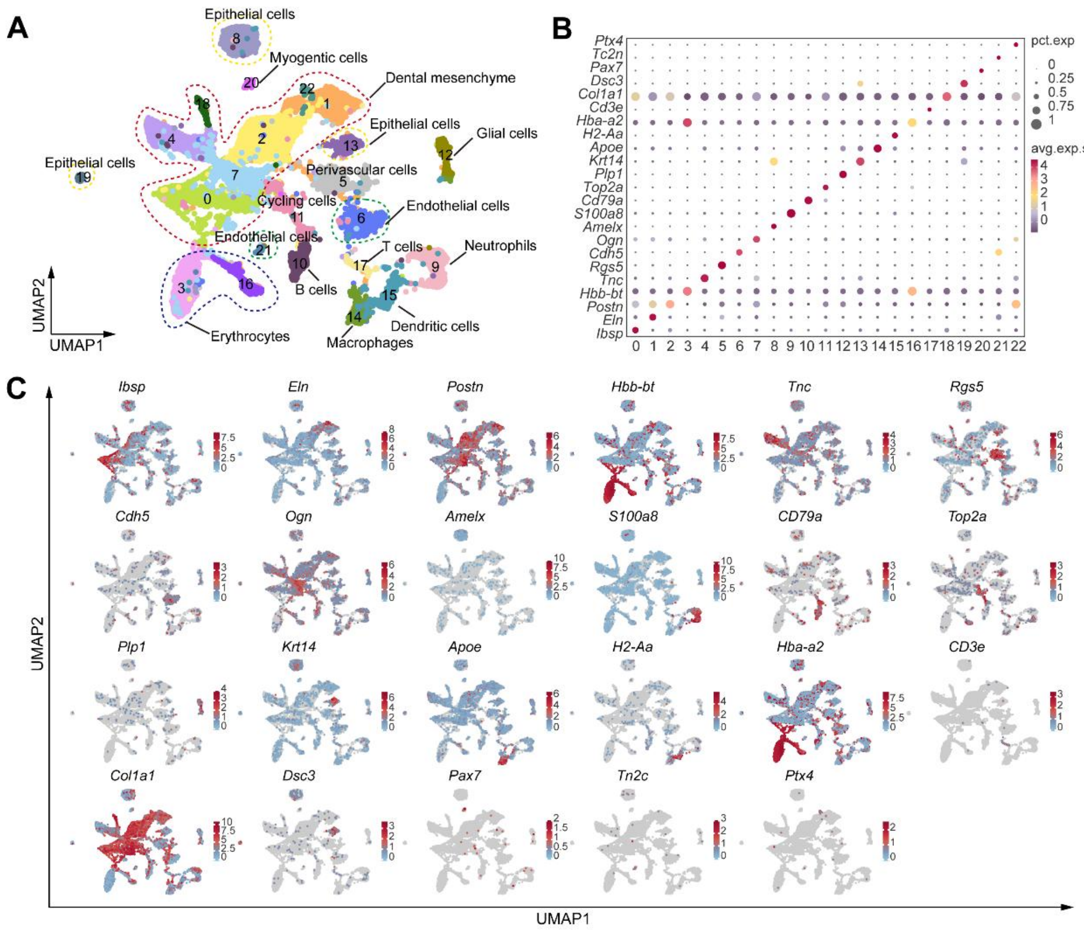

Fig. S3 Cell populations in the mouse molar and its surrounding tissue at PN15.5.

A, UMAP plot of cell types in mouse mandibular first molar and surrounding tissue at PN15.5.

B, Dot plots depicting the expression of cluster-defining genes.

C, Feature plot visualizing the expression of cluster marker genes.

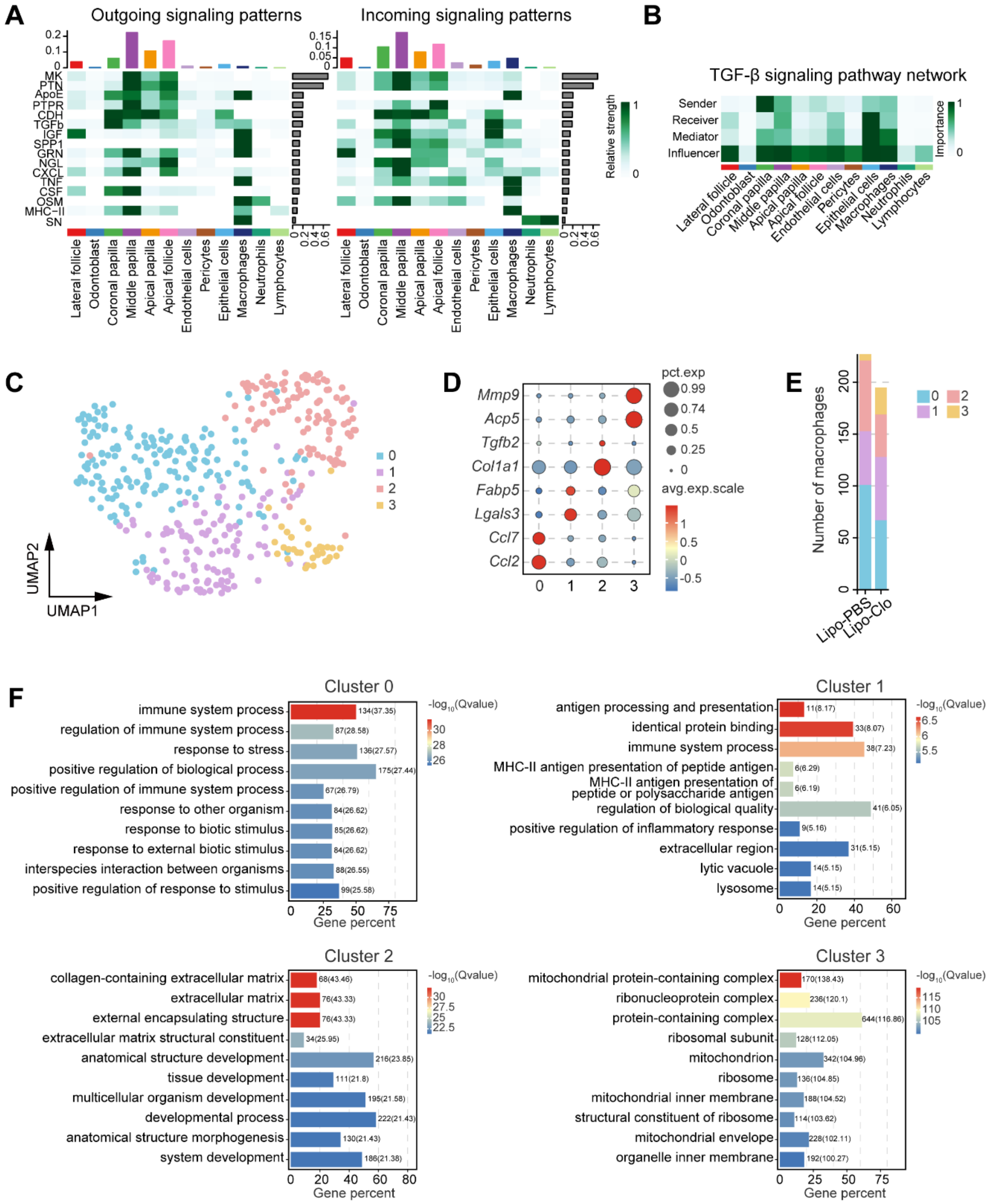

Fig. S4 Predicted cell–cell communication between macrophages and dental mesenchyme.

A, Outgoing and incoming signaling interactions among dental mesenchymal cell populations at PN3.5.

B, Inferred TGF-β signaling network between macrophages and dental mesenchymal cells at PN3.5.

C, UMAP visualization of tooth macrophages identified by scRNA-seq.

D, Dot plot showing cluster-defining marker genes across macrophage subclusters.

E, Depletion of macrophages by clodronate liposomes alters the number of macrophage subpopulations in molar.

F, Enriched GO terms in macrophage subset-specific gene profiles.

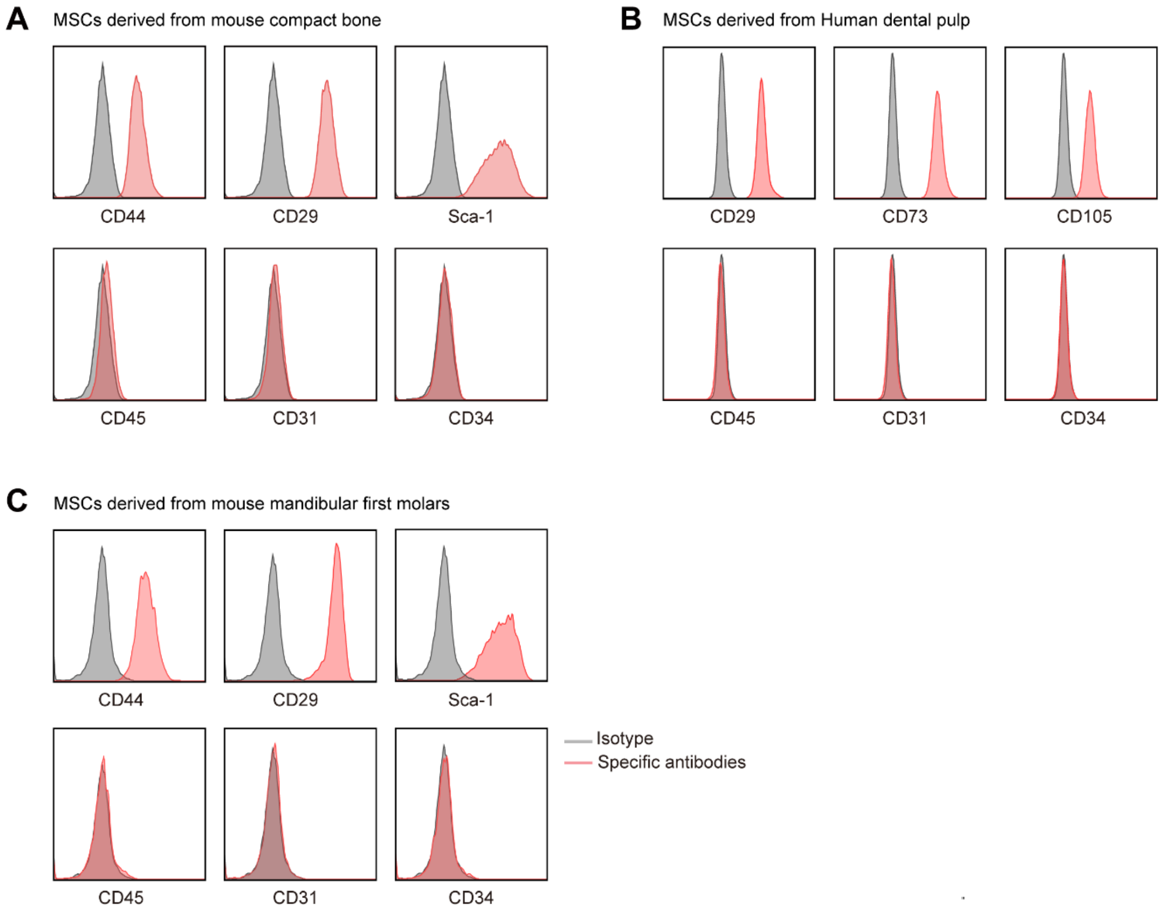

Fig. S5 Isolated primary MSCs exhibit typical MSC characteristics *in vitro*.

A, FACS analysis of cultured compact bone-derived MSCs indicates high expression of CD44, CD29 and Sca-1, but negativity for CD45, CD31, and CD34.

B, FACS analysis of cultured human dental pulp derived hDPSCs indicates high expression of CD29, CD73, and CD105, but negativity for CD45, CD31, and CD34.

C, FACS analysis of cultured mouse molar derived MSCs indicates high expression of CD44, CD29 and Sca-1, but negativity for CD45, CD31, and CD34.
