# Supplementary material for: Depletion of macrophages during early postnatal development leads to disrupted tooth root development and altered Gli1⁺ MSC trajectory
Source: Cell Death Dis. 2026 Apr 26;17(1):555. doi: 10.1038/s41419-026-08753-7 (PMC13247052; doi:10.1038/s41419-026-08753-7)
Supplement: Supplementary file 2 — Supplementary Table1 [file 41419_2026_8753_MOESM2_ESM.docx]

**Supplementary Table**

| **Reagent or resource** | **Source** | **Identifier** |
| --- | --- | --- |
| **Antibodies** |  |  |
| Anti-CD31 Monoclonal Antibody, PE, eBioscience™ (1:200) | Invitrogen | 12-0311-82 |
| Anti-CD45 Monoclonal Antibody, APC, eBioscience™ (1:200) | Invitrogen | 17-0451-83 |
| PE anti-mouse CD45 (1:200) | BioLegend | 147712 |
| APC/Cyanine7 anti-mouse CD11b (1:200) | BioLegend | 101226 |
| Brilliant Violet 421™ anti-mouse CD3 (1:200) | BioLegend | 100336 |
| PE/Cyanine7 anti-mouse F4/80 (1:200) | BioLegend | 157308 |
| Brilliant Violet 510™ anti-mouse LY6G (1:200) | BioLegend | 127633 |
| BD Horizon™ Fixable Viability Stain 700 (1:1000) | BD Biosciences | 564997 |
| CD29 Monoclonal Antibody, PE, eBioscience™ (1:200) | Invitrogen | 12-0291-82 |
| PE anti-mouse CD44 (1:200) | BioLegend | 103023 |
| PE anti-mouse Ly-6A/E (Sca-1) (1:200) | BioLegend | 108108 |
| Anti-CD73 Monoclonal Antibody, PE, eBioscience™ (1:200) | Invitrogen | 12-0731-81 |
| PE anti-mouse CD105 (1:200) | BioLegend | 120408 |
| PE anti-mouse CD34 (1:200) | BioLegend | 128610 |
| Rat IgG2b kappa Isotype Control, PE, eBioscience™ (1:200) | Invitrogen | 12-4031-81 |
| Anti-CD68 antibody FA-11 (1:200) | BioLegend | 137001 |
| Anti-F4/80 antibody BM8 (1:200) | BioLegend | 123101 |
| Anti-MHC II antibody M5/114.15.2 | BioLegend | 107601 |
| Anti-Ly6G antibody 1A8 (1:200) | BioLegend | 127601 |
| Anti-CD44 antibody IM7 (1:200) | BioLegend | 103001 |
| Anti-CD206 antibody C068C2 (1:100) | BioLegend | 141701 |
| Anti-Collagen I Polyclonal, Tm-169 (1:400) | Standard Bio tools | 3169023D |
| Rat anti-F4/80 (1:200) | Abcam | ab6640 |
| Rabbit anti-CD68 (1:200) | Cell Signaling Technology | 9778S |
| Rat anti-Ly6G(Gr-1) (1:200) | Merk | MABF474 |
| Rabbit anti-CD3 (1:200) | Cell Signaling Technology | 78588 |
| Rabbit anti-Periostin (1:200) | Abcam | ab14041 |
| Rat anti-Nestin (1:200) | Abcam | ab81462 |
| Rabbit anti-Ki67 (1:200) | Abcam | ab16667 |
| Col1a1 (E8F4L) XP rabbit mAb (1:1000) | Cell Signaling Technology | 72026S |
| RUNX2 (D1L7F) Rabbit Monoclonal Antibody (1:1000) | Cell Signaling Technology | 12256S |
| Rabbit Anti-Sp7-Osterix [EPR21034] | Abcam | ab209484 |
| Hsp90 β Rabbit mAb (1:1000) | Selleck | F1132 |
| anti-Rabbit IgG, HRP-linked Antibody (1:4000) | Cell Signaling Technology | 7074S |
| Goat anti-Rat IgG H&L (Alexa Fluor® 555) (1:1000) | Abcam | ab150158 |
| Goat anti-Rabbit 647 IgG H&L (Alexa Fluor™ Plus 647) (1:1000) | Invitrogen | A48285 |
| DAPI (1:2000) | Abcam | C1002 |
| **Critical commercial assays** |  |  |
| TRAP/ALP stain kit | Wako | 294-67001 |
| FastPure Cell/Tissue Total RNA Isolation Kit V2 | Vazyme | RC112-01 |
| 2% Alizarin Red S solution | Beyotime | C0138 |
| RNAscope Multiplex Fluorescent Reagent Kit v2 | Advanced Cell Diagnostics | 323100 |
| RNA-Protein Co-Detection Ancillary Kit | Advanced Cell Diagnostics | 323180 |
| Probe-Mm-Gli1 | Advanced Cell Diagnostics | 311001 |
| Probe-Mm-Dspp | Advanced Cell Diagnostics | 448301 |
| TSA Vivid Fluorophore 570 | Advanced Cell Diagnostics | PG-323272 |
| Maxpar® X8 Antibody Labeling Kit | Standard Biotools | 201142A |
| TCEP solution, pH 7.0 (10x1 mL,0.5 M) | MilliporeSigma | 646547 |
| Antibody Stabilizer | CANDOR® Bioscience | 130050 |
| Lymphoprep™ | StemCell Technologies | 18061 |
| EasySep™ Human CD14 Positive Selection Kit II | StemCell Technologies | 17858 |
| **Chemicals, peptides, and recombinant proteins** |  |  |
| Tamoxifen | Sigma-Aldrich | T5648-5G |
| Clodronate Liposomes | Yeasen, China | 40337ES10 |
| Control Liposomes(PBS) | Yeasen, China | 40338ES10 |
| M-CSF | GenScript | Z02930-50 |
| Human M-CSF | GenScript | Z02914-1 |
| Mouuse IL-4 Protein | NOVUS | NBP2-35131 |
| Collagenase I | Gibco | A1048301 |
| Dispase Ⅱ | Roche | 4942078001 |
| Ascorbic acid | Sigma | A8960 |
| β-glycerophosphate | Sigma | G9422 |
| Dexamethasone | Sigma | D4902 |
| SB525334 | Selleck | S1476 |
| Anti-human/mouse TGF-β-InVivo | Selleck | A2113 |
| Mouse IgG1 isotype control-InVivo | Selleck | A2106 |
