# Supplementary figures and images for: Depletion of macrophages during early postnatal development leads to disrupted tooth root development and altered Gli1⁺ MSC trajectory
Source: Cell Death Dis. 2026 Apr 26;17(1):555. doi: 10.1038/s41419-026-08753-7 (PMC13247052; doi:10.1038/s41419-026-08753-7)

# Original Data

**Fig.5B**

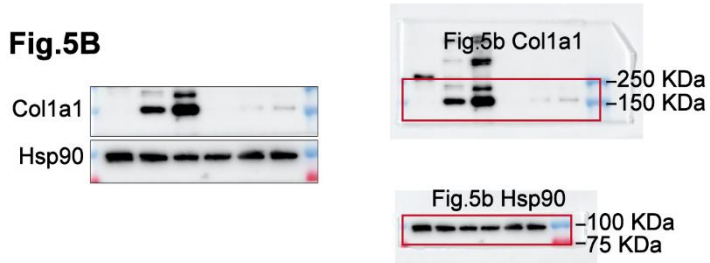

**Fig.5D**

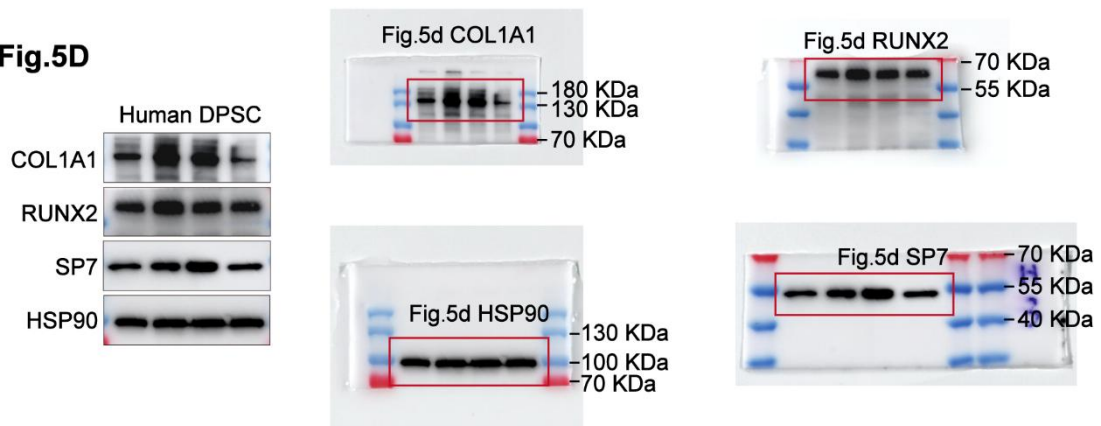

**Fig.5E**

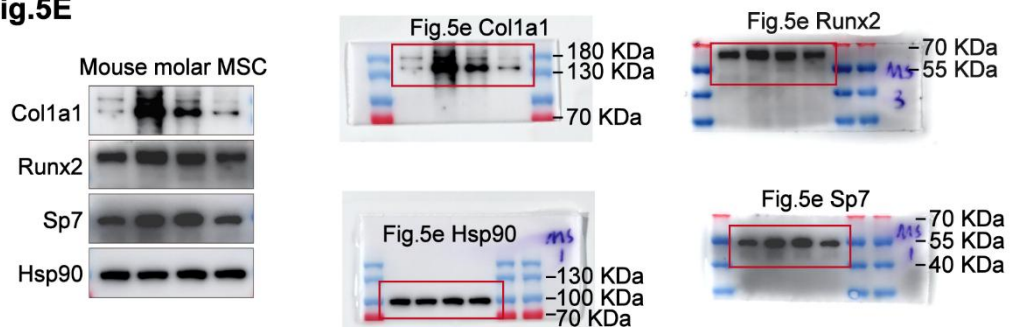

Supplement: Supplementary file 3 — Original Data [file 41419_2026_8753_MOESM3_ESM.pdf]
